# A 12‐week Exercise Intervention Among Older Adults With Chronic Coronary Syndrome: Changes and Associations of Movement Behaviors and Cardiovascular Risk Factors

**DOI:** 10.1002/ejsc.70208

**Published:** 2026-06-20

**Authors:** Antje Ullrich, Martin Bahls, Lisa Voigt, Stephanie Könemann, Marcus Dörr, Susanne Wurm, Sabina Ulbricht

**Affiliations:** ^1^ Department SHIP‐KEF Institute for Community Medicine University Medicine Greifswald Greifswald Germany; ^2^ German Centre for Cardiovascular Research (DZHK) Partner Site Greifswald Greifswald Germany; ^3^ Department of Internal Medicine B University Medicine Greifswald Greifswald Germany; ^4^ Department of Prevention Research and Social Medicine Institute for Community Medicine University Medicine Greifswald Greifswald Germany

**Keywords:** accelerometry, brain‐derived neurotrophic factor, cardiovascular marker, chronic coronary syndrome, physical activity, sedentary behavior

## Abstract

Physical activity (PA) is key to improve the prognosis of patients with chronic coronary syndrome (CCS). This study investigated changes in movement behaviors (MB), obesity markers, cardiorespiratory fitness (VO_2peak_), cardiometabolic risk (CMR), and brain‐derived neurotrophic factor (BDNF) and whether changes in MB are associated with VO_2peak_, CMR, and BDNF from baseline to 9 months after a 12‐week exercise intervention. In total, 35 CCS patients (*M* = 69.5 ± 6.4 years; 82.9% men) underwent a group‐based intervention; including two 1‐h exercise sessions per week Data was collected on anthropometrics, blood samples, cardiopulmonary exercise testing, and 7‐day accelerometry at baseline, 3‐month, and 12‐month follow‐up. Absolute changes were examined using paired *t*‐tests. Mixed‐effects linear regression models were applied to investigate associations of MB with VO_2peak_, CMR, and BDNF over time. Intra‐class correlations were calculated to estimate within‐person variability. From baseline to 3‐month follow‐up, light PA (23.7 min/d ± 10.9) increased and body mass index (−0.3 kg/m^2^ ± 1.2), glucose (−0.8 mmol/L ± 0.3), and diastolic blood pressure (−2.7 mmHg ± 1.3) decreased. From baseline to 12‐month follow‐up, there were no changes in any marker. Regression models revealed no associations between any measure of habitual movement behaviors and VO_2peak_, CMR, and BDNF over time. To conclude, short‐term but no long‐term changes in MB and cardiovascular health markers were observed among CCS patients after a 12‐week exercise intervention. Modeling the associations between MB and VO_2peak_, CMR, and BDNF over time revealed considerable within‐person variability in the data, which should be considered in future research.

**Trial Registration:**

NCT06178263, Retrospectively registered (20/12/2023)

## Highlights

- Current guidelines recommend physical activity as an important component of prevention for patients with cardiovascular disease. To date, there is scarce and inconsistent evidence on changes in various cardiovascular health markers such as movement behavior including physical activity and indicators of sedentary behavior, obesity, cardiometabolic risk, cardiorespiratory fitness or biochemical processes after a 12‐week supervised exercise intervention, especially in the long term.
- In the present study, older adults with chronic coronary syndrome were observed at baseline, immediately afterward and 9 months after a 12‐week exercise intervention. Changes and associations between movement behavior and obesity markers, cardiorespiratory fitness, cardiometabolic risk and levels of brain neurotrophic factor concentration over time were investigated.
- Our results showed short‐term but no long‐term changes in cardiovascular health markers in patients with chronic coronary syndrome after the 12‐week exercise intervention. Modeling of associations over time revealed considerable within‐person variability in the data, which should be considered in future studies.

## 1 Introduction

Individuals with cardiovascular diseases are characterized by low levels of moderate‐to‐vigorous physical activity (MVPA) and a high amount of sedentary behavior (SB) including longer periods of sitting time interrupted with less frequent breaks throughout the day compared to healthy individuals (Bakker et al. 2021; Spartano et al. 2022; Ten Broeke et al. 2022; Bellettiere et al. 2019; Jordan et al. 2023). This pattern of movement behaviors as well as a low cardiorespiratory fitness (Yue et al. 2022; Kaminsky et al. 2022), have been shown to adversely influence the prognosis of cardiovascular diseases, for example, by worsening cardiovascular and cardiometabolic parameters as well as increasing cardiovascular and all‐cause mortality (Jeong et al. 2019; Moholdt et al. 2008; Stamatakis et al. 2019; Stewart et al. 2017; Bell et al. 2022; Anderson et al. 2016; Vasankari et al. 2021).

Current guidelines recommend an active lifestyle as an important part for the prevention of cardiovascular diseases (Pelliccia et al. 2021; Valenzuela et al. 2023). In line with this, approximately 30%–70% of cardiac rehabilitation programs include physical activity as a core component (Avila et al. 2020). Although there is a plethora of evidence that the prognosis of cardiovascular diseases can be largely improved by reducing risk factors such as physical inactivity and sedentarism (Vasankari et al. 2021; Kemps et al. 2019; Visseren et al. 2021), individuals often return to being inactive shortly after the end of an exercise intervention program (Avila et al. 2020; ter Hoeve et al. 2015). Therefore, it remains a key challenge to engage patients to become more physically active and to reduce sitting time in the long term (Kahlert 2015; Hopstock et al. 2021).

Studies that included assessments of habitual movement behaviors, particularly measures of SB (Bakker et al. 2021; Ten Broeke et al. 2022; van Bakel et al. 2023), markers of obesity, cardiorespiratory fitness, and cardiometabolic risk with a follow‐up longer than 6 months after completion of an exercise intervention are scarce (Valenzuela et al. 2023). In the present study, we followed patients with chronic coronary syndrome (CCS), as one of the most common cardiovascular diseases with high prevalence worldwide (Knuuti et al. 2019), over time and measured various cardiovascular health markers from baseline to 9 months after a 12‐week supervised exercise intervention. Furthermore, we considered a biochemical marker (brain‐derived neurotrophic factor; BDNF), as there is evidence that BDNF may play a role in mediating the association between physical activity and cardiovascular health (Pius‐Sadowska and Machaliński 2017; Wang et al. 2022). However, those few studies that examined long‐term associations between cardiovascular health markers often focus on statistical analysis of mean differences between participants, which do not provide information regarding the variability within an individual to change over time (Kahlert 2015).

The primary aim of the present study was to investigate short‐term (baseline to 3 months) and long‐term changes (3–12 months, baseline to 12 months) in habitual movement behaviors (different intensities of physical activity, sitting measures), obesity markers (body mass index, BMI; waist circumference), cardiorespiratory fitness (peak oxygen uptake; VO_2peak_), cardiometabolic risk factors (blood pressure, blood lipids, glucose), and BDNF after a 12‐week multicomponent supervised exercise intervention among patients with CCS. Secondary aims were (i) to examine associations of changes in habitual movement behaviors with VO_2peak_, cardiometabolic risk (CMR), and BDNF and (ii) to quantify the extent to which within‐person variability plays a role in modeling the associations between movement behaviors and the above‐mentioned cardiovascular health markers.

## 2 Methods

### 2.1 Participants and Study Design

Inpatients of the cardiology ward at the University Medicine Greifswald were asked to participate in a pragmatic trial. The inclusion criteria for the study were defined as follows: age of at least 60 years, established CCS defined by a stenosis of more than 70% in at least one coronary vessel, and optimal medical treatment according to the guidelines of the European Society of Cardiology. Exclusion criteria included heart failure with a left ventricular ejection fraction of less than 40%, implanted cardioverter/defibrillator or pacemaker, recent cardiovascular event within 2 months prior to study inclusion (including acute myocardial infarction, resuscitation, re‐vascularization, device implantation, or stroke), planned coronary revascularization, uncontrolled blood pressure (i.e., systolic blood pressure of ≥ 200 mmHg), BMI more than 35 kg/m^2^, baseline cardiopulmonary exercise test results precluding safe exercise training (e.g., ischemia or arrhythmias), no ability to participate in exercise training (e.g., COPD GOLD III‐IV, claudication ≥ 2b, or previous disabling stroke), current mental disorder requiring inpatient treatment, current addictions (excluding tobacco use), florid psychoses, current severe depressive episode (according to the ICD‐10), severe cognitive or physical impairment, serious co‐existing diseases (e.g., cancer) with life expectancy less than 1 year, and weekly self‐reported MVPA of 150 min or more within the last 6 months prior to study inclusion. Further information on inclusion and exclusion criteria and recruitment procedure are published elsewhere (Ullrich et al. 2022). Eligible patients with CCS (*N* = 164) were invited by mail to participate in a study that aimed to assess the feasibility of a non‐controlled intervention which included a 12‐week multicomponent supervised exercise intervention. Among those who had been offered participation, 37 gave written informed consent. In total, two individuals were excluded from data analysis due to severe illness (*n* = 1) or no supervised exercise intervention attendance (*n* = 1), whereas the latter was defined as a prespecified measure of adherence (Nelson et al. 2022). Therefore, the final sample comprised 35 CCS patients. The study was conducted between November 2017 and September 2019.

**FIGURE 1:**
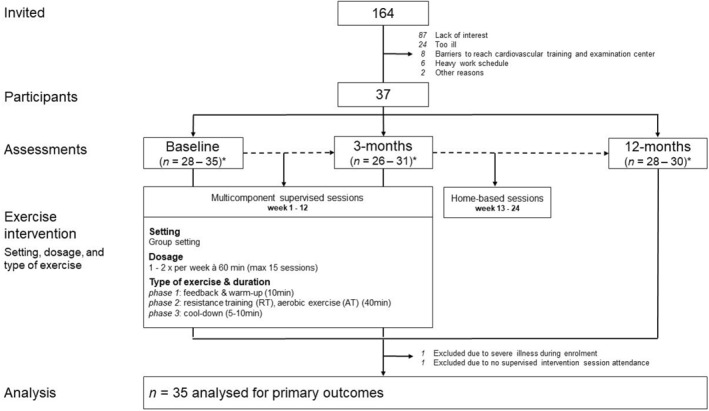
Flow chart of study participation and characteristics of the 12‐week multicomponent supervised exercise intervention. * The sample size for the three measurement points refers to the final analysis sample size of 35 participants.

At baseline, participants received a standardized assessment of anthropometrical data, blood sample taking, cardiopulmonary exercise testing, self‐administered questionnaires, and 7‐day accelerometry at the cardiovascular training and examination center of the University Medicine Greifswald. The same procedure was carried out after the 12‐week multicomponent supervised exercise intervention (3‐month follow‐up) as well as one year after baseline (12‐month follow‐up; Figure 1).

This study was conducted in accordance with the Declaration of Helsinki, current guidelines of good clinical practice, and in line with CONSORT guidelines for pragmatic trials (Zwarenstein et al. 2008). Study participants were included if they provided written informed consent. The ethics committee of the University Medicine Greifswald approved the study protocol (number BB138/17).

### 2.2 Characteristics of the Intervention Protocol

As shown in Figure 1, the exercise intervention consisted of group‐based instructor‐led multicomponent training (week 1–12) at a cardiovascular training and examination center, as well as unsupervised, self‐administered training at home (week 13–24).

An essential part of the exercise intervention was to provide information about the relevance of PA (patient education) as well as motivating participants to integrate PA into their everyday lives in order to encourage long‐term changes in their movement behavior (i.e., consultations about health behavior changes). As part of the first 12 weeks, participants were divided into five groups with seven individuals each and were provided a maximum of 22 intervention sessions; including a maximum of 15 multicomponent supervised exercise group sessions in the training center with each session lasted 60 min. From weeks 2–5, patients exercised twice per week (=8 sessions) and from weeks 6–12 once per week (=7 session). Starting at week 6, patients were instructed and motivated to exercise at home for a second home‐based, unsupervised training session. Despite the exercise sessions from weeks 1–12, additional elements of the intervention were six educational group sessions among which four sessions focused on healthy eating and two sessions on views of aging (Wurm et al. 2017). Further, there was one individual session aiming to receive process information on participants' motivation, study quality, and potential for optimization of the study. From weeks 13–24, patients exercised at home instead of group‐based supervised exercise sessions in the training center, but had the option of exercising once a week under supervision at the cardiovascular examination center (Ullrich et al. 2022). Furthermore, patients were encouraged to keep a self‐report log diary to record their exercise sessions.

### 2.3 Procedures and Measures

#### 2.3.1 Blood Samples

Study participants provided venous fasting (> 8 h) or non‐fasting blood samples which were taken between 7:30 a.m. and 1:00 p.m. Date and time of blood sampling was recorded. After sampling, the serum tubes were cooled down to 4°C. An hourly transport to the Institute for Clinical Chemistry and Laboratory Medicine of the University Medicine Greifswald was organized where the samples were immediately stored at −80°C in the Integrated Research Biobank (LiCONiC, Lichtenstein).

Plasma triglycerides, high‐density lipoprotein cholesterol, and glucose were determined by standard methodology. The samples of the resting serum levels of BDNF were diluted 1:20 with the appropriate buffer. According to the instructions of the manufacturer (Quantikine Human Free BDNF ELISA kit, R&D systems, Minneapolis, MN), serum BDNF concentrations were measured in duplicates using enzyme‐linked immunosorbent assay (ELISA) kit. The minimum detectable dose of human free BDNF using this assay is less than 20 pg/mL. The intra‐assay coefficient of variability varies between 3.8% and 6.2% while the inter‐assay precision is between 7.6% and 11.3%. Serum BDNF levels were calculated using a standard curve supplied by the ELISA kit (Ullrich et al. 2022). Trained and certified research staff performed blood sampling based on established standard operating procedures.

#### 2.3.2 Anthropometrical and Blood Pressure Data

Trained research staff measured height, weight, waist circumference, and blood pressure. Systolic and diastolic blood pressure were assessed from three readings at the right arm in a seated position using a digital blood pressure monitor (705IT, Omron Corporation, Tokyo, Japan), with a minimum resting period of three minutes. The means of the second and third reading, respectively, were used for further analysis. Waist circumference was measured midway between the lowest rib and the iliac crest using an inelastic tape. BMI was calculated as body weight in kilogram divided by height in meters squared (kg/m^2^) and used as a continuous variable in statistical analysis models of BDNF, as there seems to be an association between BMI and BDNF (Lommatzsch et al. 2005).

#### 2.3.3 Cardiometabolic Risk

CMR was expressed as a clustered CMR score, which was calculated using six continuous cardiometabolic variables that comprise the criteria for the metabolic syndrome according to the NCEP/ATP III (Grundy et al. 2004): glucose, triglycerides, high‐density lipoprotein cholesterol, waist circumference, systolic blood pressure, and diastolic blood pressure. Glucose, triglycerides, and high‐density lipoprotein cholesterol were log‐normalized. All six variables were standardized by using sex‐specific sample means (*M*) and standard deviations (SD). The CMR score was calculated by summing all standardized values and dividing the sum by six. A higher CMR score indicates a higher cardiometabolic risk (Dempsey et al. 2020; Knaeps et al. 2018).

#### 2.3.4 Cardiorespiratory Fitness

VO_2peak_ was assessed on a cycle ergometer (Ergoselect 100; Ergoline, Bitz, Germany) via standardized cardiopulmonary exercise testing according to a modified Jones protocol (Jones et al. 1985; Koch et al. 2009). Respiratory gas exchange variables were continuously measured using a MasterScreen CPX system (CareFusion, Hoechberg, Germany) and VO_2peak_ was defined as the highest ten‐second average of oxygen uptake in the last minute of exercise relative to body mass (mL/kg/min; milliliters of oxygen per kilogram body weight per minute).

Prior to the exercise test, participants were instructed to achieve maximal exhaustion. After a three‐minute rest phase, the test was started with a load of 20 W which was increased stepwise by 16 W in one hit at the end of each minute. The exercise test was carried out as symptom‐limited and terminated by the participant due to exhaustion. At the end of the test, perception of exertion was assessed using the BORG's RPE scale (Borg 2004). All exercise tests were performed by certified study staff based on established standard operating procedures (Koch et al. 2009).

#### 2.3.5 Accelerometer‐Measured Physical Activity and Sedentary Behavior

Movement behaviors were assessed using a tri‐axial accelerometer (ActiGraph GT3X + model; Pensacola, USA). Study participants were instructed to wear the accelerometer on their right hip for 10 days during waking hours, except for water activities (e.g., showering, swimming). Using ActiLife software (Actigraph Corp., Pensacola, USA), accelerations were recorded at a sampling frequency of 30 Hz and in 10 s resolution. Raw data was downloaded and 10 s data was collapsed to 60 s epochs; movement intensity was expressed as counts‐per‐minute (cpm). Data from the vertical axis was used. Non‐wear time was defined as time segments of ≥ 60 consecutive minutes of continuous zero activity counts, allowing for 2 min of counts between 0 and 100. Cut‐points for movement intensities according to threshold criteria were used: values < 100 cpm were defined as SB, values between 100 and 1951 cpm as light physical activity (LIPA), and values ≥ 1952 cpm as MVPA (Freedson et al. 1998). The mean daily time spent in MVPA, LIPA, and SB was calculated as total minutes of MVPA per day (min/d), as total minutes of LIPA per day (min/d), and as total minutes of SB per day (min/d), respectively. In addition, sitting bouts with a length of > 10‐to‐30 min and with a length of > 30 min was analyzed according to their (i) mean daily number and (ii) mean bout duration. Furthermore, mean daily time wearing the accelerometer (min/d) was obtained.

Among the 10‐day accelerometry monitoring period, only seven consecutive days of accelerometer wearing were considered. Data of the first, second, and last day of accelerometer monitoring was not included due to missing values and to minimize measurement bias of reactivity (Baumann et al. 2018). For the data from days three to nine, a wearing protocol was determined that required a minimum daily accelerometer wear time of ≥ 10 h, whereby the wear time had to exceed this predefined threshold on ≥ 4 days per week. To calculate MVPA, LIPA, and SB parameters, only data from days with predetermined sufficient wearing hours were considered as valid days and were used for the analysis to ensure consistency of data quality.

### 2.4 Statistical Analysis

Participant characteristics were described in *M* with SD. To represent changes, mean absolute changes with standard error (SE) and *p*‐values from mean‐comparison paired *t*‐tests were calculated by subtracting the first from the second value, respectively, at the following measurement points: (i) baseline to 3‐month follow‐up (Change^Baseline/3 months^), (ii) 3‐month to 12‐month follow‐up (Change^3 months/12 months^), and (iii) baseline to 12‐month follow‐up (Change^Baseline/12 months^).

Separate mixed‐effects linear regression models were analyzed to examine associations between changes in habitual movement behaviors measures (min/d of SB, LIPA, MVPA; daily mean number and length of > 10‐to‐30 min and > 30 min SB bouts), and VO_2peak_, CMR, and BDNF, respectively. The continuous exposures were mean‐centered to facilitate the interpretation. Sex as a nominal variable and age as a continuous variable were assessed by a self‐administered questionnaire. Adherence to a physical exercise intervention, as an important factor in increasing the potential benefits related to this program (Ruano‐Ravina et al. 2016), was included as a discrete variable in statistical analysis. Thus, all regression models were adjusted for sex, age, multicomponent supervised exercise intervention attendance, and accelerometer wear time. All models that included physical activity variables (i.e., MVPA and LIPA) were additionally adjusted for time spent in SB. All models including SB measures were additionally adjusted for MVPA. Finally, all models with the outcome variable BDNF were additionally adjusted for BMI.

Analyses were conducted using all available data from participants who enrolled and started the interventions assuming data was missing at random. Furthermore, we used non‐parametric bootstrapping (*b* = 1000 repetitions) to account for the small sample size (Efron and Tibshirani 1994). In each model, a two‐way interaction of exposure × time (1 = baseline; 2 = 3‐month; 3 = 12‐month) was included to test the differential effect of each exposure over time. We used robust standard errors estimations to account for heteroscedasticity. All models were estimated using the mixed command (and the *bootstrap* prefix) in STATA (StataCorp LLC, Texas, USA, Version: 17.0). *p*‐values < 0.05 were considered statistically significant.

To investigate within‐person variability, we calculated intra‐class correlations (ICC) by dividing the between‐person variance by the sum of within‐ and between‐person variances. The ICC ranged between 0 and 1 and can be interpreted as the correlation between repeated measurements within the same cluster (=individual) (Liljequist et al. 2019). A larger ICC means a higher correlation within the cluster; it thus indicates a lower variability within a cluster and a higher variability between the clusters. In order to compare different regression models and to estimate the fit of the model to a different sample (=reproducibility), we calculated the Corrected Akaike Information Criteria [AICc] to account for the small sample size (Konishi and Kitagawa 2008), with lower values indicating a better model fit.

## 3 Results

### 3.1 Characteristics of Study Participants

At baseline, the mean age of participants was 69.5 years (SD = 6.4), 82.9% (*n* = 29) were male, and 6.1% (*n* = 4) were current smokers. In total, 40% (*n* = 14) attended school less than 10 years, 20% (*n* = 7) 10 years, and 40% (*n* = 14) more than 10 years. Overall, 88.6% (*n* = 31) reported a vascular intervention (e.g., stent), 100% received antihypertensives, 91.4% statins (*n* = 32), 22.9% antidiabetics (*n* = 8), and 8.6% antidepressants (*n* = 3). The mean number of the attended supervised multicomponent supervised exercise intervention sessions was 12.5 (SD = 3.2; min: 1, max: 15). Table 1 shows descriptive characteristics of the study sample separated for baseline, 3‐month, and 12‐month follow‐up.

**TABLE 1:** Characteristics of the study sample (*n* = 35) separated for baseline, 3‐month follow‐up, and 12‐month follow‐up.

Parameters	Baseline *n* = 28–35^a^	After 3 months *n* = 26–31^a^	After 12 months *n* = 28–30^a^
Glucose, mmol/L	7.5 ± 2.7	6.4 ± 2.0	7.1 ± 3.1
HDL‐C, mmol/L	1.2 ± 0.3	1.2 ± 0.3	1.3 ± 0.3
Triglycerides, mmol/L	1.7 ± 1.1	1.7 ± 1.2	1.8 ± 1.5
Systolic blood pressure, mmHg	128.4 ± 17.8	130.9 ± 14.3	125.7 ± 12.2
Diastolic blood pressure, mmHg	68.9 ± 7.0	66.6 ± 7.0	68.5 ± 6.3
Waist circumference, cm	102.4 ± 12.4	100.9 ± 12.1	102.2 ± 14.8
Body mass index, kg/m^2^	29.4 ± 5.1	28.6 ± 4.4	29.5 ± 5.6
Accelerometer wear time, min/d	792.4 ± 75.7	809.5 ± 85.1	800.4 ± 73.1
Valid days of wearing, 7 days	18 (56.3)	14 (46.7)	17 (53.1)
Light physical activity, min/d	261.5 ± 70.5	290.5 ± 70.6	259.7 ± 56.9
Moderate‐to‐vigorous physical activity, min/d	21.6 ± 15.4	26.0 ± 18.5	21.1 ± 15.0
Sedentary time, min/d	509.2 ± 77.8	493.0 ± 63.2	519.6 ± 78.7
Bouts analysis of sedentary behavior (> 10–30 min)			
Mean number per day	15.6 ± 3.2	14.8 ± 2.7	16.1 ± 2.5
Mean bout length per day (min)	22.1 ± 2.2	21.6 ± 2.5	21.8 ± 2.6
Bouts analysis of sedentary behavior (> 30 min)			
Mean number per day	3.4 ± 1.2	3.4 ± 1.7	3.6 ± 1.6
Mean bout length per day (min)	43.9 ± 2.4	42.6 ± 2.8	42.8 ± 2.6
VO_2peak_, mL/kg/min	20.9 ± 5.2	22.0 ± 5.2	20.7 ± 5.2
Cardiometabolic risk score	−0.0 ± 0.4	−0.0 ± 0.4	0.0 ± 0.5
Brain‐derived neurotrophic factor, ng/mL	48.5 ± 10.3	49.4 ± 9.5	49.5 ± 12.3

*Note:* Data are presented as *n* (%) for categorical variables and mean (*M*) ± standard deviation (SD) for continuous variables, respectively.

Abbreviations: HDL‐C = high‐density lipoprotein cholesterol; VO_2peak_ = peak oxygen uptake.

^a^ Due to missing data, sample sizes varied at the different time points.

At baseline, participants spent 64.4% of their accelerometer wear time in SB, 32.9% in LIPA, and 2.7% in MVPA (*n* = 29). At 3‐month follow‐up, participants spent 61.2% of their accelerometer wear time in SB, 35.6% in LIPA, and 3.1% in MVPA (*n* = 28). At 12‐month follow‐up, participants spent 64.8% of their accelerometer wear time in SB, 32.5% in LIPA, and 2.6% in MVPA (*n* = 28).

### 3.2 Absolute Changes in Markers of the Cardiovascular Examination Program Over Time

From baseline to 3‐month follow‐up, glucose levels (−0.8 mmol/L ± 0.3; *t* (29) = −2.7), diastolic blood pressure (−2.7 mmHg ± 1.3; *t* (30) = 0.8), and BMI (−0.3 kg/m^2^ ± 1.2; *t* (30) = −2.4) decreased and LIPA (23.7 min/d ± 10.9; *t* (22) = 2.2) increased (all *p*‐values < 0.05). Between 3‐month and 12‐month follow‐up, LIPA decreased (−37.4 min/d ± 14.2; *t* (24) = −2.6), whereas HDL‐C (0.1 mmol/L ± 0.0; *t* (26) = 2.4), SB (39.7 min/d ± 15.3; *t* (24) = 2.6), and daily mean number of > 10–30 min SB bouts (1.5 ± 0.6; *t* (24) = 2.5) increased (all *p*‐values < 0.05). No significant changes of any measures were observed from baseline to 12‐month follow‐up (Table 2).

**TABLE 2:** Absolute changes in markers of the assessments over the three measurement points.

	Change ^Baseline/3 month^ *n* = 23–31^a^				Change ^3 month/12 month^ *n* = 25–27^a^				Change ^Baseline/12 months^ *n* = 24–30^a^			
	Absolute	*df*	*t*	*p*	Absolute	*df*	*t*	*p*	Absolute	*df*	*t*	*p*
Glucose, mmol/L	−0.8 ± 0.3	30	−2.704	**0.011**	0.4 ± 0.5	25	0.850	0.403	−0.2 ± 0.3	29	−0.545	0.590
HDL‐C, mmol/L	−0.0 ± 0.0	30	−0.478	0.636	0.1 ± 0.0	26	2.409	**0.023**	0.1 ± 0.0	29	1.361	0.184
Triglycerides, mmol/L	−0.0 ± 0.2	30	−0.154	0.879	0.1 ± 0.2	26	0.574	0.571	0.0 ± 0.2	29	0.218	0.829
Systolic blood pressure, mmHg	2.5 ± 3.2	30	0.762	0.452	−6.3 ± 3.2	25	−1.954	0.062	−5.2 ± 3.0	28	−1.742	0.096
Diastolic blood pressure, mmHg	−2.7 ± 1.3	30	−2.064	**0.048**	1.7 ± 1.5	25	1.099	0.283	−0.7 ± 1.6	28	−0.410	0.685
Waist circumference, cm	−0.8 ± 1.1	30	−0.733	0.470	−0.9 ± 6.2	25	−0.737	0.467	−1.1 ± 1.2	28	−0.892	0.380
Body mass index, kg/m^2^	−0.3 ± 1.2	30	−2.436	**0.021**	−0.2 ± 1.0	25	−1.069	0.295	−0.4 ± 0.2	28	−1.707	0.099
Light physical activity, min/d	23.7 ± 10.9	22	2.167	**0.041**	−37.4 ± 14.2	24	−2.625	**0.015**	−12.0 ± 9.4	23	−1.276	0.215
Moderate‐to‐vigorous physical activity, min/d	7.2 ± 3.7	22	1.966	0.062	−6.8 ± 3.6	24	−1.904	0.069	−0.6 ± 2.2	23	−0.253	0.803
Sedentary time, min/d	−10.9 ± 15.5	22	−0.700	0.492	39.7 ± 15.3	24	2.588	**0.016**	5.8 ± 15.0	23	0.384	0.704
Bouts analysis of sedentary behavior (10–30 min)												
Mean number per day	−0.4 ± 0.7	22	−0.598	0.556	1.5 ± 0.6	24	2.526	**0.019**	0.5 ± 0.6	23	0.813	0.425
Mean length per bout and day (min)	−0.5 ± 0.5	22	−0.900	0.378	0.6 ± 0.5	24	1.210	0.238	−0.4 ± 0.5	23	−0.988	0.334
Bouts analysis of sedentary behavior (> 30 min)												
Mean number per day	0.1 ± 0.3	22	0.196	0.846	0.5 ± 0.3	24	1.680	0.106	0.3 ± 0.3	23	1.100	0.283
Mean length per bout and day (min)	−1.0 ± 0.8	22	−1.192	0.246	0.4 ± 0.8	24	0.473	0.640	−1.0 ± 0.6	23	−1.639	0.115
VO_2peak_, mL/kg/min	0.6 ± 0.4	29	1.331	0.193	−0.6 ± 0.4	25	−1.763	0.090	−0.2 ± 0.4	28	−0.451a	0.656
Cardiometabolic risk score	0.0 ± 0.1	29	−0.270	0.994	0.0 ± 0.1	24	0.139	0.879	0.0 ± 0.1	27	−0.052	0.810
Brain‐derived neurotrophic factor, ng/mL	1.5 ± 1.4	24	1.053	0.303	−0.8 ± 1.7	25	−0.431	0.670	0.2 ± 1.8	26	0.099	0.744

*Note:* Data are presented as mean (*M*) ± standard error (SE) for continuous variables. Bold = statistically significant with *p*‐values < 0.05 based on paired *t*‐tests.

Abbreviations: HDL‐C = high‐density lipoprotein cholesterol; VO_2_peak = peak oxygen uptake.

^a^ Due to missing data, sample sizes varied at the different time points.

### 3.3 Associations of Habitual Movement Behaviors With Cardiorespiratory Fitness, Cardiometabolic Risk, and Brain‐Derived Neurotrophic Factor

Using bootstrapped data, results of the mixed‐effects linear regression models revealed no significant association of any habitual movement behaviors measures with VO_2peak_, CMR, and BDNF over time (Table 3). The correlations among the values of the three repeated measurements within the same cluster differed between the outcome variable (VO_2peak_, CMR, BDNF); with ICC values between 0.48 and 0.50, 0.62 and 0.67, as well as 0.90 and 0.91, respectively. To compare and estimate the reproducibility of the different regression models, information criteria values were calculated. The lowest fit indices values (AICc) were found in regression models including sitting measures with bouts lasting longer than 30 min (VO_2peak_: “Mean length of > 30 min of SB bouts per day”, CMR and BDNF: “Number of > 30 min of SB bouts per day”).

**TABLE 3:** Estimated coefficients, confidence intervals (95%), intraclass correlation coefficients, and information criteria for VO_2peak_, cardiometabolic risk, and brain‐derived neurotrophic factor using separate mixed‐effects linear regression models of bootstrapped data.

		Outcomes								
		VO_2peak_ (*n* = 34)^a^			CMR (*n* = 33)^a^			BDNF (*n* = 29)^b^		
Exposures		b (95% CI)	ICC	AICc	b (95% CI)	ICC	AICc	b (95% CI)	ICC	AICc
LIPA										
Exposure x time (ref. Baseline)	3 months	−0.01 (−0.03, 0.01)	0.90	448.96	−0.00 (−0.00, 0.00)	0.49	179.05	−0.02 (−0.10, 0.07)	0.64	553.94
	12 months	−0.01 (−0.03, 0.01)			0.00 (−0.00, 0.01)			0.07 (−0.03, 0.17)		
Intercept		16.90 (11.13, 22.67)			0.06 (−0.52, 0.65)			44.90 (22.28, 67.51)		
MVPA										
Exposure x time (ref. Baseline)	3 months	−0.00 (−0.08, 0.08)	0.90	445.10	0.00 (−0.01, 0.02)	0.51	178.98	−0.12 (−0.46, 0.22)	0.64	549.57
	12 months	0.00 (−0.08, 0.08)			0.00 (−0.02, 0.03)			0.19 (−0.25, 0.64)		
Intercept		16.49 (10.72, 22.26)			0.04 (−0.56, 0.64)			47.27 (27.03, 67.50)		
SB										
Exposure x time (ref. Baseline)	3 months	0.00 (−0.01, 0.02)	0.90	450.98	−0.00 (−0.00, 0.00)	0.50	183.53	0.02 (−0.06, 0.10)	0.62	555.15
	12 months	0.00 (−0.01, 0.02)			−0.00 (−0.01, 0.00)			−0.05 (−0.14, 0.05)		
Intercept		16.49 (10.68, 22.29)			0.02 (−0.58, 0.63)			44.65 (23.42, 65.88)		
SB bout Nr > 10‐30										
Exposure x time (ref. Baseline)	3 months	0.05 (−0.40, 0.51)	0.90	432.93	0.03 (−0.06, 0.11)	0.50	164.05	0.69 (−1.10, 2.49)	0.67	536.18
	12 months	0.13 (−0.41, 0.66)			−0.03 (−0.17, 0.10)			−1.15 (−3.61, 1.30)		
Intercept		16.19 (10.25, 22.14)			0.10 (−0.58, 0.77)			42.26 (18.82, 65.70)		
SB bout Nr > 30										
Exposure x time (ref. Baseline)	3 months	0.44 (−0.53, 1.40)	0.90	428.95	−0.01 (−0.20, 0.18)	0.49	160.60	0.52 (−3.77, 4.81)	0.63	531.55
	12 months	0.24 (−0.69, 1.17)			−0.09 (−0.30, 0.12)			−2.55 (−7.11, 2.01)		
Intercept		15.84 (10.07, 21.60)			0.03 (−0.53, 0.59)			43.60 (21.59, 65.61)		
SB bout length > 10‐30										
Exposure x time (ref. Baseline)	3 months	0.38 (−0.22, 0.97)	0.90	429.45	−0.03 (−0.14, 0.07)	0.50	164.46	−0.35 (−2.45, 1.75)	0.63	533.19
	12 months	0.05 (−0.49, 0.60)			−0.04 (−0.15, 0.07)			−1.96 (−4.56, 0.65)		
Intercept		15.63 (10.33, 20.93)			0.04 (−0.49, 0.57)			43.91 (22.66, 65.15)		
SB bout length > 30										
Exposure x time (ref. Baseline)	3 months	0.26 (−0.36, 0.89)	0.91	426.89	−0.03 (−0.12, 0.07)	0.51	164.31	−1.75 (−4.34, 0.84)	0.66	533.22
	12 months	−0.30 (−0.87, 0.27)			0.02 (−0.10, 0.13)			−1.78 (−4.41, 0.85)		
Intercept		15.44 (9.88, 21.01)			0.11 (−0.45, 0.68)			40.84 (16.94, 64.73)		

Abbreviations: AICc = Corrected Akaike's information criterion, *b* = unstandardized regression coefficient, BDNF = Brain‐derived neurotrophic factor, CI = Confidence Interval, ICC = intraclass correlation coefficients, Interaction term = exposure × time, LIPA = Light physical activity, MVPA = Moderate‐to‐vigorous physical activity, Ref = Reference, SB = Sedentary behavior, VO_2peak_ = peak oxygen uptake.

^a^ Adjusted for sex, age, accelerometer wear time, intervention adherence, and for SB in the regression models that included physical activity variables (LIPA, MVPA); regression models including SB were adjusted for MVPA.

^b^ Adjusted for sex, age, body mass index, accelerometer wear time, intervention adherence, and for SB in the regression models that included physical activity variables (LIPA, MVPA); regression models including SB were adjusted for MVPA.

## 4 Discussion

The present study shows data on movement behaviors, obesity markers, cardiorespiratory fitness, cardiometabolic risk, and a biochemical marker (BDNF) assessed at three measurement time points in patients with CCS who participated in a 12‐week multicomponent exercise intervention. The study revealed two main findings. First, there were short‐term absolute changes in some CCS‐related markers (3‐month follow‐up) but no long‐term changes (12‐month follow‐up). Second, no statistically significant associations between any measure of habitual movement behaviors and VO_2peak_, CMRS, and BDNF over time were present. The extent of within‐person variability varied between the regression models.

In line with previous studies (Kaminsky et al. 2022; Dibben et al. 2021; Marzolini et al. 2012), our data revealed changes in the habitual movement behaviors directly after the 12‐week supervised exercise intervention. A small but statistically significant increase of time spent in LIPA (0.4 h/day) was found. Given the current evidence that any intensity of physical activity has substantial health benefits (including LIPA) (Ekelund et al. 2019; Dempsey et al. 2020), this change indicates a favorable modification in the movement behaviors directly after the completion of our supervised exercise intervention. So far, studies showed reductions in overall sitting time per day, whereas no changes in daily MVPA were observed after supervised cardiac rehabilitation (Bakker et al. 2021; Ten Broeke et al. 2022). We found a decrease in BMI (−0.3 kg/m^2^), levels of glucose (−0.8 mmol/L), and diastolic blood pressure (−2.7 mmHg) at 3‐month follow‐up, which is in line with a study showing changes in obesity markers (e.g., weight, waist circumference) and cardiometabolic risk factors (e.g., blood pressure, blood lipids) among 16 at‐risk individuals for cardiovascular diseases directly after a 6‐month intervention (Hopstock et al. 2021).

As shown in similar intervention studies, these changes seem to be temporarily and individuals often return to their previous movement behaviors within months after participating in an exercise program (ter et al. 2015.; Hopstock et al. 2021; Dibben et al. 2021; Nicolini et al. 2019). At 12‐month follow‐up, no statistically significant changes were observed in any marker of movement behaviors, indicating a lack of long‐term maintenance of our 12‐week multicomponent supervised exercise intervention. Recently, findings of a 12‐week personalized SB intervention embedded in cardiac rehabilitation showed favorable absolute changes in time spent in SB, LIPA, and MVPA as well as a reduction in cardiovascular risk between pre‐ and post‐rehabilitation in the intervention and in the control group. However, the changes between both groups did not differ significantly. The authors explain this by features of the study design (e.g., lack of blinding of treatment) that may have led to cross‐over effects from the intervention to the control group (van Bakel et al. 2023). Given that interventions aimed to reduce sedentarism in patients with cardiovascular diseases are scarce, further research in this patient group is warranted.

To interpret the results of this study, it is important to compare the sample characteristics with those from similar intervention studies (Bakker et al. 2021; Ten Broeke et al. 2022; van Bakel et al. 2023). Compared to these, individuals with CCS in our study spent less time sedentary, more time in LIPA, less time in MVPA, and had fewer prolonged SB bouts (≥ 30 min/d). Although our study participants were approximately seven years older and predominantly male (∼+8%), baseline characteristics of movement behaviors suggest that our study sample already had an active lifestyle with less sedentarism prior to the beginning of the 12‐week exercise intervention. Indeed, 41.4% of our study participants met the recommendations of the World Health Organization of ≥ 150 min of MVPA per week (Bull et al. 2020) and the percentage of those who were at‐risk for detrimental health effects from sitting time ≥ 9.5 h/d was small (17.2%) (Ekelund et al. 2019). An important methodological difference between the studies has to be noted. In our study, we used hip‐worn devices (ActiGraph GT3X+), while the three other studies used thigh‐worn devices (actiPAL3) to assess movement behaviors. There is evidence that the accuracy of the estimates is rather low when comparing both devices, although the differences appear to be small at the group level using the vertical axis only (Pfister et al. 2017).

So far, studies have often focused on the statistical analysis of the mean values across time without considering within‐person variability of movement behaviors and their associations with cardiovascular and cardiometabolic risk factors (Kahlert 2015). In fact, this study adds to the literature that while there are no statistically significant associations between measures of habitual movement behaviors and VO_2peak_, CMR, and BDNF over time, there are noticeable differences in within‐person variability of the results. Based on the ICC values, the lowest variability of repeated measures within the same individual was found in the regression models of VO_2peak_ (0.90–0.91). A modest within‐person variability was found in the regression models of CMR (0.49–0.51) and BDNF (0.62–0.67). The highest correlation of measurements in regression models with cardiorespiratory fitness as the outcome might be explained by study design features. In patients with cardiovascular diseases, medium‐interval high‐intensity interval training seems to be more effective than moderate‐intensity continuous training for increasing cardiorespiratory fitness, with recommendations for training sessions at least three times per week with a duration between 7 and 12 weeks (Yue et al. 2022; Hannan et al. 2018). Due to the fact that this pragmatic trial was designed to examine the feasibility and adherence to an intervention program in a usual care setting (Zwarenstein et al. 2008), not to increase specific cardiorespiratory fitness markers, and therefore to inform the development of further studies, the intervention duration and dosage were rather modest.

In fact, evidence is still lacking on how a supervised exercise intervention should be designed to allow patients with cardiovascular diseases to maintain favorable modifications in their health profile. Our study results suggest that the within‐person variability of the change in risk factors for cardiovascular disease progression by physical exercise training is modest to high. Therefore, the physical capacity and baseline movement behaviors prior to program start might be considered to estimate the effectiveness of an intervention, but also the trajectory of each individual's changes during the intervention (Kemps et al. 2019; Kambic et al. 2023), as this offers the potential to adapt the intervention over time. In line with this proposal, current guidelines recommend tailored exercise programs for cardiovascular disease patients with different demographical and disease‐specific background (Pelliccia et al. 2021; Kemps et al. 2019). In addition, the results of the present study showed that the highest reproducibility of the regression models was found with models that included movement behaviors measures for prolonged SB bouts (exposures: “number of > 30 min SB bouts”; “average length of > 30 min SB bouts”). Given that information on changes in daily SB patterns in response to a physical exercise intervention in patients with cardiovascular diseases over time are scarce, our results could provide a first impression of which SB pattern measures should be considered and analyzed in future studies. However, future research should emphasize how and to what extent within‐person variability and the reproducibility of measures of movement behaviors should be considered in the design, individualized adaption of interventions, and data analysis of physical exercise intervention in patients with cardiovascular diseases.

A major strength of this study was the inclusion of long‐term follow‐up to examine changes in habitual movement behaviors and various cardiovascular health markers after a 12‐week multicomponent supervised exercise intervention in individuals with CCS. Assuming that a physically active lifestyle is related with long‐term health benefits in patients with cardiovascular diseases, our study also examined associations between changes in habitual movement behaviors and cardiorespiratory, cardiometabolic, and biochemical markers, which are known in the literature as risk factors for cardiovascular disease progression. To date, few intervention studies in this patient group have (i) examined changes in risk factors of disease progression in longer‐term follow‐up, and those that did show conflicting evidence (ter Hoeve et al. 2015; Dibben et al. 2021), and (ii) considered habitual SB measures such as number and average lengths of SB bouts (Bakker et al. 2021; Spartano et al. 2022; Ten Broeke et al. 2022; van Bakel et al. 2023). Furthermore, given the low drop‐out rate and high level of adherence to the 12‐week multicomponent supervised exercise intervention sessions, we assume that our intervention was feasible and accepted by the participants.

The present study has several limitations that should be considered. First, we expect that individuals who were more interested in their health or more motivated to change their behavior more likely agreed to participate in our intervention, which could introduce sample bias (Hardcastle et al. 2015). As discussed above, the baseline characteristics of our study sample partially confirmed this assumption. Although the sampling procedure and the resulting selection bias may result in data that inadequately represent the target population and therefore cannot be generalized to all individuals with CCS, these methodological issues are well known from intervention studies in general. Second, our sample size was small and the proportion of male participants was high, which is not uncommon in the literature (Wang et al. 2022; Marzolini et al. 2012). However, the small sample size of females did not allow to stratify our data by sex which seems to be an important factor, particularly to extract the effect of long‐term physical activity on risk factors for cardiovascular disease progression (Wang et al. 2022; Safdar and Mangi 2020). Third, both fasting and non‐fasting blood samples were collected to investigate absolute changes in blood lipids, glucose, and calculate the CMR score. Although the blood lipid or glucose profiles could be influenced by external factors (e.g., caloric intake or muscle activity), evidence highlighted the advantages of non‐fasting blood sampling in using blood lipids to predict cardiovascular risk (Langsted and Nordestgaard 2019). Furthermore, the fasting status of the study participants was not recorded at the three measurement points. Potential changes in the metabolic regulation of lipid and glucose profiles due to fasting status in each patient over time could therefore not be included as covariate in the model. This is an important aspect that should be included in the design of future studies. Fourth, in collecting, preparing, and analyzing accelerometer‐based data of movement behaviors (Migueles et al. 2017), we followed guidelines for the general population because there are currently no specific recommendations to handle accelerometer‐based data of cardiovascular patients (Vetrovsky et al. 2020). Fifth, our study was designed as a pragmatic non‐controlled trial and therefore, should be interpreted cautiously with respect to the design and the results. Important confounding variables that may influence long‐term changes and associations of movement behaviors and risk factors of cardiovascular disease progression were not considered or controlled such as medication, other lifestyle behaviors, physical environment characteristics, or psychosocial factors (Ullrich et al. 2022). Furthermore, due to the lack of a control group, it is difficult to distinguish whether the results were influenced by the intervention or other factors such as placebo effects or the progression of the disease.

## 5 Conclusions

The results of the present study showed some short‐term but no long‐term changes in movement behaviors, obesity markers, cardiorespiratory fitness (VO_2peak_), cardiometabolic risk, and BDNF after a 12‐week exercise intervention in individuals with CCS. No associations could be found between any measures of habitual movement behaviors and VO_2peak_, CMR, and BDNF over time, but the data revealed substantial within‐person variability that should be considered in future research.

Although physical inactivity and levels of SB are high in older adults with cardiovascular diseases, interventions aimed at reducing sedentarism are scarce in this patient group. Future research requires the development of interventions that should (i) include components that more strongly target fragmented SB patterns and (ii) identify behavioral, psychological, and physical environmental factors that may influence the long‐term maintenance of intervention effects, such as changes in movement behaviors.

**Abbreviations**
AICC: Corrected Akaike Information Criteria
BDNF: Brain‐derived neurotrophic factor
BMI: Body mass index
CCS: Chronic coronary syndrome
CMR: Cardiometabolic risk
CPM: Counts per minute
ELISA: Enzyme‐linked immunosorbent assay
ICC: Intra‐class correlations
LIPA: Light physical activity
M: Mean
MVPA: Moderate‐to‐vigorous physical activity
SB: Sedentary behavior
SD: Standard deviation
SE: Standard error
VO_2peak_
: Peak oxygen uptake

## Author Contributions

MD, SW and SU planned und designed the study. MD and SU also managed participants' recruitment and data collection. AU and SU analyzed and interpreted the data. AU drafted the manuscript. SU supervised the writing and MB, SK, LV, MD, and SW provided additional input. All authors read, critically revised, and approved the final version of the manuscript.

## Funding

This study was supported and funded by grants from the Federal Ministry of Education and Research as part of the German Centre of Cardiovascular Research (DZHK; Grant No. D347000002). The DZHK had no direct role in the development of methodology, the acquisition, analysis, and interpretation of data or in writing the manuscript. In addition, the study was supported and funded by grants from the Research Network Community Medicine of the University Medicine Greifswald (Grant No. FOVB‐2020‐10).

## Ethics Statement

All procedures performed in studies involving human participants were in accordance with the ethical standards of the institutional and/or national research committee and with the 1964 Helsinki Declaration and its later amendments or comparable ethical standards. Further, this study was conducted in line with the current guidelines of good clinical practice and the CONSORT guidelines for pragmatic trials. The ethics committee of the University Medicine Greifswald approved the study protocol (number BB 138/17). Written informed consent was obtained before inclusion of study participants.

## Consent

All authors gave their consent for publication.

## Conflicts of Interest

The authors declare no conflicts of interest.

## Data Availability

The datasets generated and/or analyzed during the current study are not publicly available due to restrictions associated with anonymity of participants but are available from the corresponding author on reasonable request. The data is shared with researchers who submit a methodologically sound proposal to achieve the aims of the approved proposal. Requests in this regard should be directed to the corresponding author to gain access. Requestors must sign a data access agreement ensuring data usage in compliance with the statement given in the informed consent procedure and with the German data protection law, that the data will not be transferred to others, and that the data will be deleted after the intended analysis has been completed.
